# Cerebral venous sinus thrombosis caused by traumatic brain injury complicating thyroid storm: a case report and discussion

**DOI:** 10.1186/s12883-022-02777-0

**Published:** 2022-07-07

**Authors:** Shurong Gong, Wenyao Hong, Jiafang Wu, Jinqing Xu, Jianxiang Zhao, Xiaoguang Zhang, Yuqing Liu, Rong-Guo Yu

**Affiliations:** 1 Department of Surgical Critical Care Medicine, Fujian Provincial Hospital, Provincial Clinical College of Fujian Medical University, Fuzhou, China; 2 Department of Neurosurgery, Fujian Provincial Hospital, Provincial Clinical College of Fujian Medical University, Fuzhou, China

**Keywords:** Cerebral venous sinus thrombosis (CVST), Traumatic brain injury, Thyroid storm, Case report, Low molecular weight heparin (LMWH)

## Abstract

**Introduction:**

Cerebral venous sinus thrombosis (CVST) is an uncommon cerebrovascular disease with diverse predisposing factors. We report a case of CVST caused by a thyroid storm induced by traumatic brain injury.

**Case presentation:**

A 29-year-old male patient with a history of Graves’ disease with hyperthyroidism presented to our hospital with head trauma of cerebral contusion and laceration in both frontal lobes confirmed by admission CT scan. He received mannitol to lower intracranial pressure, haemostatic therapy, and antiepileptic treatment. Eight days later, he presented with signs of thyroid storms, such as tachycardia, hyperthermia, sweating and irritation, and his thyroid function tests revealed high levels of TPO-Ab, TR-Ab, TG-Ab, FT3 and FT4. Then, he entered a deep coma. His brain CT showed a thrombosis of multiple venous sinuses, along with the opening of peripheral collateral vessels, congestive infarction with haemorrhage and brain swelling. He regained consciousness after treatment with antithyroid drugs, anticoagulants, respiratory support and a regimen of sedation/analgesia. After a half-year follow-up, most of the patient’s blocked cerebral venous sinuses had been recanalized, but there were still some sequelae, such as an impaired fine motor performance of the right hand and verbal expression defects.

**Conclusions:**

CVST can be induced by thyroid storms, and trauma-related thyroid storms can develop on the basis of hyperthyroidism. The purpose of this case report is to raise clinicians’ awareness and improve their ability to diagnose CVST early in patients with traumatic brain injury complicating thyroid storms to improve the neurological prognosis among similar patients.

## Introduction

Cerebral venous sinus thrombosis (CVST) is believed to be caused by intracranial venous blood stasis, coagulation hyperactivity and venous endothelial injury. The annual incidence of CVST is up to 15.7 per million persons [1]. Due to atypical clinical manifestations, it is often easily missed or misdiagnosed. Hyperthyroidism may be one of the predisposing factors for CVST [2], but nonspecialist physicians generally have limited awareness of this condition. When a patient with hyperthyroidism is admitted to the hospital due to severe trauma, clinical interventions surrounding trauma are frequently implemented without adequate attention to hyperthyroidism, which might result in adverse consequences. Recently, we admitted a case of a thyroid storm precipitated by traumatic brain injury that resulted in CVST. This report aimed to examine the pathogenesis and regimen selection, as well as treatment and monitoring for CVST, to improve the early diagnosis and differentiation of CVST among clinicians.

## Case presentation

**Fig. 1:**
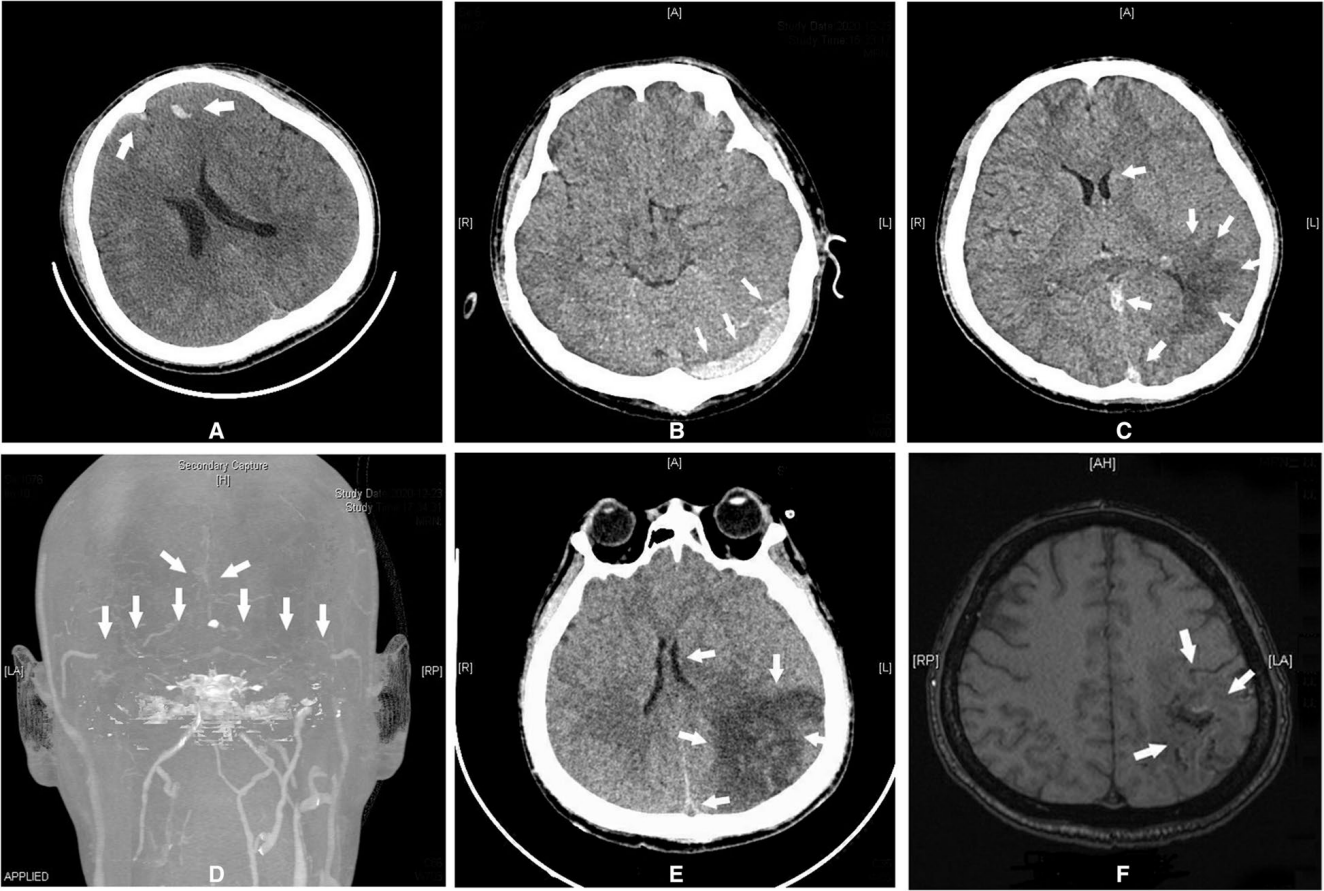
(**A-F**): **A** The initial plain CT scan on December 12 showed a cerebral contusion and laceration in bilateral frontal lobes and an intracerebral haematoma in left frontal lobe; **B** Plain CT scan on December 23 showed high-density thrombosis in the left transverse sinus; **C** Plain CT scan on December 23 revealed thrombosis in the sinus confluence and straight sinus, cerebral parenchymal infarction in the left parieto-occipital lobe, swelling of the left hemisphere, and compression of the left ventricle; **D** CT venography of December 23 showed absence of flow-related enhancement in the superior sagittal sinus, confluence of sinuses, and bilateral transverse sinuses, suggesting venous sinus thrombosis with open peripheral collateral vessels. **E** Plain CT scan on December 29 showed progression of the low-density lesion of the left parietotemporal occipital lobes and obvious brain swelling. **F** MRI on January 24 showed a patchy infarct lesion in the left parietal lobe and improvement of the surrounding brain swelling. The white solid arrows show the lesions

A 29-year-old male patient was admitted to the emergency department because of head trauma caused by a fall from a height on December 12, 2020. He suffered acute confusion for 5 minutes immediately after trauma. He presented generalized convulsions and complained of obvious dizziness and headache after waking up. Examination of the CT scan (Fig. 1A) showed signs of cerebral contusion and laceration in both frontal lobes and bilateral pulmonary contusions. After a negative screening result of a nucleic acid test for COVID-19, he was admitted to the department of neurosurgery the next day. On the admitting physical examination, he was afebrile with a regular pulse rate of 100/min and blood pressure of 162/97 mmHg. He had a clear mind and multiple abrasions on the top of the right forehead. He showed no abnormalities in the cardiopulmonary examination and received a Glasgow Coma Scale score of 15 (E4V5M6). His admission diagnosis was light cerebral contusion and laceration in the bilateral frontal lobes, intracerebral haematoma in the left frontal lobe and scalp haematoma on the right frontal scalp. Then, he received mannitol to lower intracranial pressure, haemostatic therapy, antiepileptic treatment and close monitoring for changes in consciousness and vital signs. His father provided a medical history of more than 1 year of Graves’ disease with hyperthyroidism, taking methimazole irregularly without regular review. His thyroid function tests thereby revealed a serum thyroid-stimulating hormone (S-TSH) level 0.01 mIU/L (NR: 0.27 ~ 4.2), thyroid peroxidase antibody (TPO-Ab) 288.1 IU/ml (NR: 0 ~ 34), thyrotropin receptor antibody (TR-Ab) > 40 U/L (NR: 0 ~ 1.75), thyroglobulin antibody (TG-Ab) > 4000 IU/ml (NR: 0.1 ~ 115), free triiodothyronine (FT3) 17.68 pmol/L (NR: 3.1 ~ 6.8), and free thyroxine (FT4) 69.9 pmol/L (NR: 12 ~ 22). Then, he was prescribed self-administered methimazole.

**Fig. 2:**
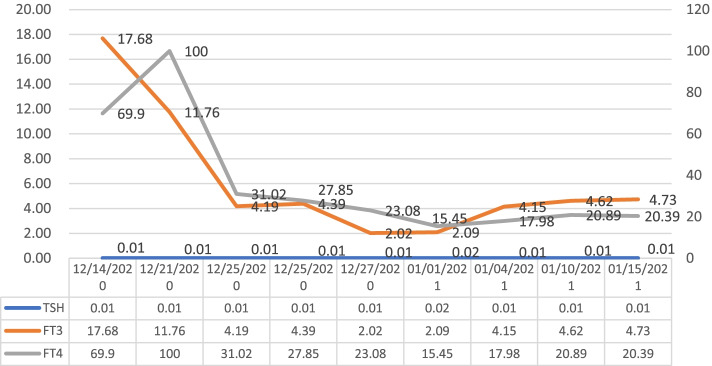
FT3 and FT4 gradually decreased with the implementation of the antihyperthyroidism treatment during the course of CVST. FT3/FT4 units: pmol/L (FT3 NR: 3.1 ~ 6.8, FT4 NR: 12 ~ 22); TSH units: mIU/L (NR: 0.27 ~ 4.2)

**Fig. 3:**
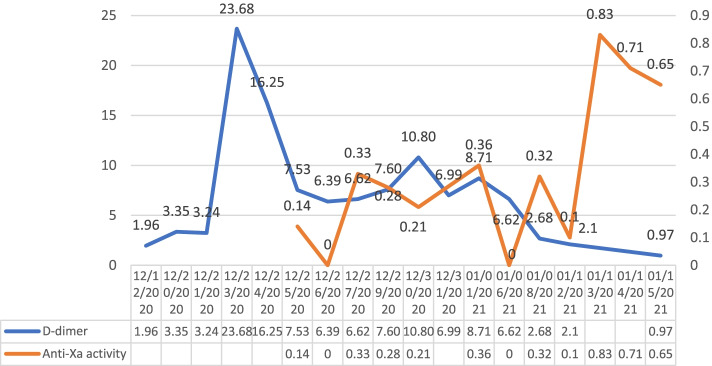
The fluctuation of plasma D-dimer and anti-Xa activity. Plasma D-dimer reached the highest point on December 23, which was the most serious moment of the disease. With the implementation of anticoagulation on December 23, the anti-Xa activity fluctuated with an increasing trend, while D-dimer gradually decreased, and the anticoagulant effect was ultimately achieved. D-dimer units: mg/L (NR: 0 ~ 0.55); anti-Xa activity units: U/ml (effective range: 0.4 ~ 1.0)

At 3 am on December 20th, the patient became irritable and was unable to respond correctly with a heart rate of 120/min. His body temperature increased to 38.5 °C, his blood pressure reached 200/120 mmHg, and he began profusely sweating. After the endocrinology consultation, he was suspected of being in a thyroid storm. Then, he was transferred to the ICU for further diagnosis and treatment. The patient continued to have a high fever up to 40 °C and a heart rate > 130/min. He endured respiratory distress and was endotracheally intubated to receive ventilator-assisted breathing. Re-examination of thyroid function tests revealed S-TSH 0.01 mIU/L (NR: 0.27 ~ 4.2), TPO-Ab 307 IU/ml (NR: 0 ~ 34), TR-Ab> 40 U/L (NR: 0 ~ 1.75), TG-Ab> 4000 IU/ml (NR: 0.1 ~ 115), FT3 11.76 pmol/L (NR: 3.1 ~ 6.8), and FT4 > 100 pmol/L (NR: 12 ~ 22). He received a Burch & Wartofsky score of 55 points [3] (≥45 points is highly indicative of a thyroid storm). Plasma catecholamine tests showed dopamine 383.3 pmol/L (NR: < 195.7), epinephrine 1164.5 pmol/L (NR: < 605.4), and norepinephrine 6600.0 pmol/L (NR: 414.0 ~ 4435.5). The patient received adrenal gland imaging scans and obtained a negative result. The patient was confirmed to be in a thyroid storm and received a series of antihyperthyroidism treatments, including propylthiouracil, iodine, propranolol, hydrocortisone and plasmapheresis. Meanwhile, he entered a deep coma (GCS 2 T scores), and re-examination of his brain CT and CTV (Fig. 1B-D) showed a thrombosis of multiple venous sinuses, along with opening of peripheral collateral vessels, congestive infarction with haemorrhage and brain swelling. His neurological condition deteriorated rapidly. According to multidisciplinary consultation from neurosurgery, neurology and endocrinology, as well as family wishes, physicians-in-charge chose a treatment protocol containing subcutaneous injection of low molecular weight heparin (LMWH) 5000 IU (approximately 100 IU/kg) q 12 h, thyroid storm control, mannitol to lower intracranial pressure, sedation and analgesia, rather than aggressive endovascular thrombectomy. The patient’s temperature and heart rate gradually decreased, and his FT3 and FT4 values also showed similar trends (Fig. 2). LMWH was injected on December 23, 2020, and the anticoagulant effect was determined by dynamically monitoring the changes in D-dimer and plasma anti-Xa activity (Fig. 3). D-dimer gradually decreased, and the level of plasma anti-Xa activity gradually reached approximately 0.4 U/ml (effective range of 0.4 ~ 1.0 in our institute). A CT scan performed on December 29th revealed that the low-density lesions in the left parietal/temporal/occipital lobes had progressed with obvious brain swelling (Fig. 1E). The patient’s coma status gradually improved, and his temperature, heart rate and blood pressure gradually returned to normal ranges. He regained consciousness on January 10th, 2021 and was gradually weaned from mechanical ventilation; then, he received physical rehabilitation. His plasma thrombosis screening revealed slightly low protein C activity and normal levels of protein S activity and homocysteine, with no lupus anticoagulant detected. His indices for autoimmune disorders showed no *abnormalities.* An MRI scan on January 24th (Fig. 1F) revealed a patchy infarct lesion in the left parietal lobe, and the surrounding brain swelling improved. The patient was discharged on January 28th and transferred to Shanghai for further treatment. His thyroid function indices were in the normal range before discharge (Fig. 2), with D-dimer decreasing to 0.48 mg/L. However, his right upper limb muscle strength remained poor. Rivaroxaban was prescribed as the main oral anticoagulant to substitute LMWH before discharge.

**Fig. 4:**
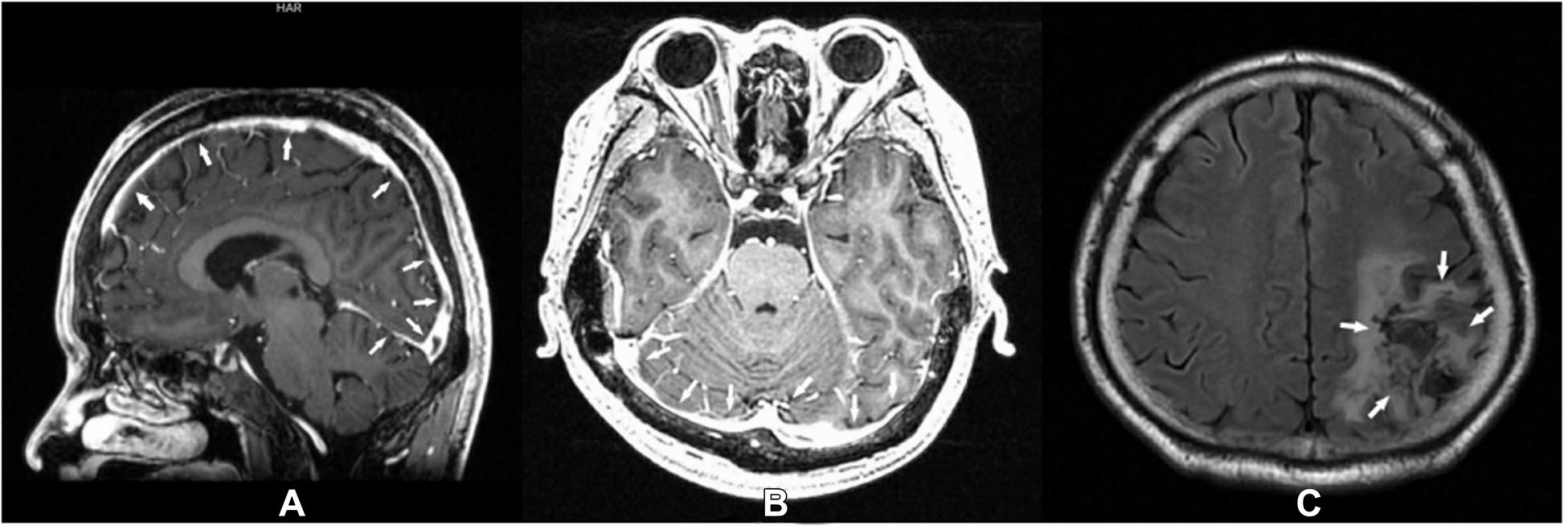
**A-C** Reexamination of MRI half a year after discharge: **A** Recanalization of the superior sagittal sinus, straight sinus, sinus confluence, and a small thrombus was still visible near the sinus confluence; **B** Good enhancement was seen in the sinus confluence and bilateral transverse sinuses, and some residual thrombus could be seen in the initial segments of the bilateral transverse sinuses; **C** MRI showed a focal lesion of encephalomalacia with gliosis in the left parieto-occipital lobes. The white solid arrows show the lesions

After discharge, the patient continued to receive treatments, including oral antihyperthyroidism (methimazole), anticoagulation (rivaroxaban), and rehabilitation exercises. Half a year after discharge, the grand movement ability and muscle strength of his right upper limb gradually recovered. However, the fine movements of his right hand were still imperfect, and his language presentation skills were slightly impaired. His MRI on July 8th revealed a focal lesion of encephalomalacia with gliosis in the left parieto-occipital lobes and recanalization of the previously blocked venous sinuses, and a small thrombus was still visible in the left transverse sinus and sinus confluence (Fig. 4A-C). His hyperthyroidism was well controlled, with an FT3 of 5.5 pmol/L (NR: 3.1 ~ 6.8) and an FT4 of 15.5 pmol/L (NR: 12 ~ 22). He continues to take oral medication and undergo physical rehabilitation.

## Discussion

Cerebral venous sinus thrombosis (CVST) is a rare thrombotic disease. Recent studies have found that its incidence may be greatly underestimated [1, 4]. The aetiology and risk factors for CVST include infectious factors, pregnancy and postpartum status, systemic disease, dehydration, intracranial tumours, oral contraceptives, hypercoagulable status, certain drugs, trauma, COVID-19, and vaccination with adenovirus-vaccines [5], and approximately 30% of CVST cases still have unknown aetiology [6, 7]. CVST caused by traumatic brain injury is rare [8, 9]. On the basis of uncontrolled hyperthyroidism and precipitated by certain factors, such as infection, trauma, and surgery, thyroid storms occur and lead to rapid deterioration, involving multiple organs and an overall mortality of up to 20–30% [10]. Severe trauma is a rare cause of thyroid storms [10–12]. It is a challenge for admitting physicians to identify thyroid storms among trauma patients because they commonly focus on dealing with significant injuries. Manifestations such as tachycardia and unconsciousness are often thought to be related to trauma. Hyperthyroidism is considered a risk factor for CVST. Hieber et al. [2] found that 20.9% of CVST patients had thyroid diseases, and the ratio was much higher than previous studies on risk factors for CVST [6, 7, 13]. A number of previous studies have also shown a correlation between hyperthyroidism and CVST [14–17] because of the hypercoagulable state induced by thyrotoxicosis [18, 19]. Thyrotoxicosis increases plasma levels of tissue factor, Factor VIII, Factor IX, von Willebrand Factor, fibrinogen, D-dimer, and plasminogen activator inhibitor-1 [20, 21], these factors are all related to the formation of CVST.

This patient with poorly controlled hyperthyroidism received mannitol to lower intracranial pressure, haemostasis and antiepileptic treatment after brain trauma. He was instructed to take antihyperthyroidism drugs by himself without surveillance. He developed a thyroid storm and a coma 8 days after admission, and his CT re-examination scan showed multiple cerebral venous sinus thromboses and progressive congested cerebral infarction of the left parieto-occipital lobe with brain swelling, which led to a deep coma. Witnesses stated that the patient likely lost consciousness before falling, which meant a hyperthyroidism-related brain condition might have existed before trauma. During the episode of his thyroid storm, we found an interesting phenomenon: the patient had a high level of catecholamine, which indicates sympathetic hyperactivity. Hyperthyroidism can affect a physiologic state similar to catecholamine excess, leading to a series of manifestations [22]. On the other hand, catecholamines increase T4-to-T3 conversion in selected tissues, showing a synergistic interaction between thyroid hormone and the sympathetic system [23]. In previous studies [24, 25], plasma and urinary levels of norepinephrine in thyrotoxicosis patients have been reported as either normal or diminished. Interestingly, we proposed an opposite result in our case. We could not fully confirm the aetiology of sympathetic hyperactivity after adrenal gland imaging scans, but we obtained a good therapeutic effect after antihyperthyroidism plus antisympathetic therapy (propranolol). We believe that the thyroid storm enhanced sympathetic activity in this case, and there was still a complicated interaction between these two systems. More evidence is needed to support our inference. According to the above analysis, this patient developed a trauma-inducing thyroid storm on the basis of poorly controlled hyperthyroidism and ultimately developed multiple CVSTs and cerebral infarction. Under a series of intensified treatments, the patient’s situation was gradually under control, and he slowly regained consciousness. During his follow-up, long-term anticoagulant and hyperthyroidism control became routine therapy because his hyperthyroidism is a predisposing factor of thrombosis, and his conditions are currently well controlled. The lessons learned from this case are as follows: well-controlled hyperthyroidism should not be ignored under any situation, and careful investigation of the medical history and increasing awareness of trauma-associated thyroid storms may help reduce the misdiagnosis rate and prevent catastrophic consequences.

The 2017 European Stroke Organization guideline for the diagnosis and treatment of CVT [26] recommended the use of LMWH as the main treatment for acute CVT. The United States guidelines suggested endovascular therapy considered in patients with clinical deterioration despite anticoagulation or with severe neurological deficits or coma [27]. Currently, endovascular therapy is not recommended as a routine treatment for CVST. In this case, the decision-maker was reluctant to accept the risks of endovascular therapy; therefore, anticoagulation with LMWH became a reasonable choice in the situation of a thyroid storm. However, there were still some sequelae left due to multiple CVST leading cortex infarctions. In the future, if there are more reliable manoeuvres of endovascular treatment, the neurological prognosis of such patients will likely be improved.

There is currently no recommendation for testing anti-Xa activity to monitor the effectiveness of LMWH [28, 29] because the treatment range of LMWH might have no association with clinical results. However, certain groups of patients may benefit from this monitoring, including pregnant women, children, obese patients, and patients with renal impairment [29]. The tendency of a gradual increase in anti-Xa activity corresponded to the gradual decline in D-dimer in this patient (Fig. 3). However, the anti-Xa activity curve showed a vast fluctuation, which meant it could only be used as a reference instead of complete guidance for treatment.

Our case report has some limitations. We could not provide direct evidence from trauma to CVST, and there might have been some preexisting pathologic status in his brain before falling. We could not fully rule out other aetiologies of sympathetic hyperactivity during his thyroid storm. We did not complete a full screen for all related thromboembolic disease, haematological diseases, oncological diseases or rheumatological diseases, which might have been the underlying aetiology for CVST. We did not have enough safe endovascular manoeuvres to choose from in order to minimize subsequent cerebral sequelae other than anticoagulation. We will handle similar cases better in the future with the accumulation of experience.

## Conclusion

This report describes a case of CVST caused by traumatic brain injury complicated by thyroid storm with the goal of improving our understanding of the diagnosis and treatment of similar cases. We propose the following learning points:

- ▼ If a patient with traumatic brain injury has underlying hyperthyroidism, the admitting physicians should pay attention to the severity of hyperthyroidism and maintain close monitoring to prevent thyroid storm development.
- ▼ CVST can be induced by a thyroid storm. Timely craniocerebral imaging should be performed when a stable patient with hyperthyroidism develops cognitive impairment or even coma during treatment.
- ▼ Currently, anticoagulation is the most reasonable choice for CVST. If technical conditions are allowed, aggressive endovascular therapy could be considered to reduce neurological damage and sequelae [27].
- ▼ Monitoring of anti-Xa activity can be considered for patients who are prone to secondary bleeding or unaffordable on bleeding consequences (such as cerebral haemorrhage).

## Data Availability

All data are available in the manuscript.

## Abbreviations

NR: Normal range
LMWH: Low molecular weight heparin
CVST: Cerebral venous sinus thrombosis
